# The Effects of rTMS Combined with Motor Training on Functional Connectivity in Alpha Frequency Band

**DOI:** 10.3389/fnbeh.2017.00234

**Published:** 2017-11-29

**Authors:** Jing-Na Jin, Xin Wang, Ying Li, Fang Jin, Zhi-Peng Liu, Tao Yin

**Affiliations:** ^1^Institute of Biomedical Engineering, Chinese Academy of Medical Sciences & Peking Union Medical College, Tianjin, China; ^2^Neuroscience Center, Chinese Academy of Medical Sciences, Beijing, China

**Keywords:** repetitive transcranial magnetic stimulation, motor training, phase synchronization index, functional connectivity, graph theory

## Abstract

It has recently been reported that repetitive transcranial magnetic stimulation combined with motor training (rTMS-MT) could improve motor function in post-stroke patients. However, the effects of rTMS-MT on cortical function using functional connectivity and graph theoretical analysis remain unclear. Ten healthy subjects were recruited to receive rTMS immediately before application of MT. Low frequency rTMS was delivered to the dominant hemisphere and non-dominant hand performed MT over 14 days. The reaction time of Nine-Hole Peg Test and electroencephalography (EEG) in resting condition with eyes closed were recorded before and after rTMS-MT. Functional connectivity was assessed by phase synchronization index (PSI), and subsequently thresholded to construct undirected graphs in alpha frequency band (8–13 Hz). We found a significant decrease in reaction time after rTMS-MT. The functional connectivity between the parietal and frontal cortex, and the graph theory statistics of node degree and efficiency in the parietal cortex increased. Besides the functional connectivity between premotor and frontal cortex, the degree and efficiency of premotor cortex showed opposite results. In addition, the number of connections significantly increased within inter-hemispheres and inter-regions. In conclusion, this study could be helpful in our understanding of how rTMS-MT modulates brain activity. The methods and results in this study could be taken as reference in future studies of the effects of rTMS-MT in stroke patients.

## Introduction

Motor training (MT) can influence cortical activity related to motor task, and is a major means of improving limb movement function, especially in people with motor dysfunction, such as stroke patients (Van et al., 2001; Higgins et al., 2006). However, the motor functional outcomes induced by MT often have limitations. Transcranial magnetic stimulation (TMS) is a non-invasive brain stimulation method that can alter cortical excitability via application of time-varying magnetic field. Repetitive TMS (rTMS) is one TMS pattern that produces serial outputs of TMS stimuli pulses. rTMS can modulate cortical activity. The changes in cortical activity can persist long after the end of stimulation (Thut and Pascual-Leone, 2010) to influence motor and cognitive functions (Baeken et al., 2012; Turriziani et al., 2012; D'Agata et al., 2016). Therefore, rTMS has been proposed as a potential method to improve motor functions in stroke patients (Fregni et al., 2006; Conforto et al., 2012; Brodie et al., 2014).

Recently, a majority of studies has found the combination protocol of rTMS-MT to improve motor functions of the paretic upper limb to an extent that was unattainable by either rTMS or MT alone (Bolognini et al., 2009; Emara et al., 2010; Avenanti et al., 2012; Kakuda et al., 2012, 2016; Lüdemann-Podubecká et al., 2015). Avenanti et al. (2012) studied the long-term behavioral effect of rTMS-MT in chronic stroke, and found that receiving MT prior to the rTMS was optimal in boosting functional outcomes. Furthermore, although there were three combination patterns for rTMS to improve motor functional outcomes, including downregulation of excitability by low frequency rTMS over contralesional hemisphere (≤1 Hz), upregulation of excitability by high frequency rTMS over lesional hemisphere (≥5 Hz), and bihemispheric regulation by low frequency rTMS over contralesional hemisphere associated with high frequency rTMS over the lesional hemisphere, the first one was the most popular method used in stroke patients. One reason for this choice might be that low frequency rTMS was safer and had lower probability of epileptic complication. Another reason might be that the effects of low frequency rTMS stimulation of the contralesional hemisphere on brain activity was slightly affected by changes in the brain induced by stroke.

Although many studies have shown that rTMS-MT could improve motor performance in stroke patients, the impacts of rTMS-MT on brain activity remain to be fully understood. Takekawa et al. (2014) studied the effects of rTMS-MT on regional brain perfusion using single-photon emission computed tomography (SPECT) in chronic stroke patients, and found changes in asymmetry index in the superior and middle frontal areas, which correlated significantly and negatively with changes in Fugl-Meyer assessment (FMA) score. In another study, the authors measured F-wave parameter to evaluate the impact of rTMS-MT on motor neural excitability (Kondo et al., 2015). However, studies that tested the effects of rTMS-MT on functional network are scarce.

The human cerebral cortex exhibits specific functional interconnection patterns that link all brain regions. The linking patterns are neither completely regular nor random, i.e., the human brain integrates both localized and segregated information processing (Sporns et al., 2004), and can be modulated by circumstances and external stimuli. A number of studies have demonstrated that rTMS protocols could alter the activity and function of targeted brain region as well as its related remote regions (Jing and Takigawa, 2000; Plewnia et al., 2008; Grefkes et al., 2010). The activity of many cortices related to motor function, such as premotor, primary motor, and posterior parietal cortex can also be changed by MT (De Vico Fallani et al., 2017; Youssofzadeh et al., 2016). Instead of local regional activity, functional connectivity, which can be obtained through analysis of inter-regional coupling, reflects the functional interactions between the underlying brain regions (Ward and Cohen, 2004; Grefkes et al., 2010). Therefore, we can speculate that rTMS and MT could modulate brain functional connectivity. However, there are limited studies that have analyzed the modulation of rTMS-MT on functional connectivity.

Brain activity in the resting state with eyes closed is the baseline status of the human brain that is independent of task-related brain functions. The study of resting state neural activity is an alternative approach to task related regional activity and likely provides additional information on the neurobiological mechanisms of specific brain systems (Fox and Raichle, 2007; Van Dijk et al., 2010). Neural oscillation at alpha frequency band (8–13 Hz) is the main rhythm in the resting state and constitutes an important neural substrate for cognition and action. This oscillation is supposed to be able to predict the efficiency of neural cognitive-motor processes when the subject is involved in demanding tasks, in eyes closed resting state condition (Babiloni et al., 2010). Thus, it is critical to study the changes of alpha band neural activity in the resting state in order to understand brain responses induced by rTMS-MT.

Studies of complex human brain networks using graph theory as a mathematical model have attracted much interest in recent years (Stam and Reijneveld, 2007; Stern, 2013). This approach consists of a set of elements (nodes) and connections (edges) that interconnect the nodes of the graph. In our study, each node was represented by an electroencephalography (EEG) electrode and the edges by functionally connected brain regions. This approach can be used to examine changes in functional architectural connectivity of the brain, which result from diseases and external stimuli (Ward and Cohen, 2004; Grefkes et al., 2010). The results of recent studies suggest that functional disconnection between distant brain areas could at least partly explain many brain dysfunctions, such as Alzheimer's disease (AD) and stroke (Stam et al., 2007a,b). Therefore, brain functional connectivity and graph theoretical analysis may be combined to achieve a better insight into the impact of rTMS-MT on cortical functions.

In order to understand the impact of rTMS-MT on brain activity, we recruited healthy subjects and evaluated whether functional cortical architecture at alpha frequency band in the resting state changed. To achieve this, we first tested their behavioral performances before and after rTMS-MT. Then, we recorded 60-channels EEG before and after rTMS-MT in healthy volunteers who were in resting state with eyes closed. Correlation matrices were based on calculation of the phase synchronization index (PSI) between all pair wise combinations of the 60 EEG channels and subsequently undirected graphs were built. Finally, we calculated the relationship between functional connectivity and behavioral data by Pearson's correlation. Our methods and results could be helpful in future for studying the effects of rTMS-MT in stroke patients.

## Materials and methods

### Subjects

The study involved 10 healthy volunteers (10 men; mean age 23.4 ± 2.1 years). None of them suffered from any significant neurological disorder or had any implanted metallic electrical devices or took any medication in the 2 weeks before their participation in the experiment. All subjects were right handed according to the Edinburgh handedness inventory. The study was performed according to the Declaration of Helsinki and approved by the Ethics Committee of Institute of Biomedical Engineering, Chinese Academy of Medical Sciences & Peking Union Medical College. All subjects provided informed consent prior to inclusion in the study.

### Procedure

In our study, the purpose of rTMS-MT was to improve the motor activity of non-dominant hand. The main reason was that, compared with dominant hand, the non-dominant hand was not usually used in daily day and couldn't perform lots of movements flexibly. We selected three MTs which were unfamiliar to non-dominant hand, and detected the improvement of non-dominant hand induced by rTMS-MT. In other words, the non-dominant hand of healthy person was analogied to the lesional-hand of stroke in our study.

**Figure 1:**
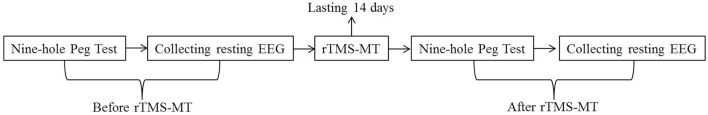
Experimental paradigm. Low frequency rTMS was first delivered to the dominant hemisphere, followed by training of the non-dominant hand, which lasted 14 days. Nine-Hole Peg Test and resting state EEG with eyes closed were recorded before and after rTMS-MT.

In general, the rTMS-MT was based on a model of interhemispheric competition model in stroke (Farzan et al., 2010). Up-regulating excitability of the lesioned motor cortex or down-regulating excitability in the intact motor cortex could improve the motor performance. Therefore, in our study, in order to achieve improved motor functions of the non-dominant hand, low frequency rTMS was first delivered to the dominant hemisphere to downregulate its excitability, followed by training of the non-dominant hand intended to upregulate the excitability of the non-dominant hemisphere (Avenanti et al., 2012). Figure 1 shows the experimental paradigm. The subjects performed rTMS-MT for 14 days. The Nine-Hole Peg Test was performed to assess the dexterity of bilateral hands. The subjects had to complete the Nine-Hole Peg Test as quickly as possible. The reaction time was recorded, and resting EEG with eyes closed was obtained before and after rTMS-ML.

### Repetitive transcranial magnetic stimulation

rTMS was delivered through a figure-eight shaped coil (70 mm standard coil, Mastic, Whiteland, UK) connected to a Mastic Rapid2 stimulator (Mastic, Whitland, UK). In order to precisely stimulate the target brain area (primary motor cortex), we used a frameless stereotactic neuro-navigation system (Brainsight, Rogue Inc., UK). All stimulation was applied over the left primary motor cortex (dominant hemisphere). The coil was placed tangentially to the scalp with the handle pointing backwards and laterally at a 45° angle away from the midline. The primary motor cortex was individually localized for each participant based on the optimal position for eliciting motor-evoked potentials (MEPs) in the right abductor pollicis brevis (APB). The individual resting motor threshold (RMT) was defined as the minimum stimulus intensity that could produce an MEP of at least 50 μV in at least five out of 10 consecutive trials.

Each subject received 10 sessions of rTMS at a frequency of 1 Hz over the 14 days, excluding Sundays. The stimulation target was the left hemisphere over the primary motor cortex corresponding to the “hot spot” for stimulation of the APB cortical area representation as defined during RMT determination. One rTMS session consisted of 1,200 pulses, lasting 20 min. The intensity of stimulation was set at 90% RMT.

### Motor training

The MT consisted of 3 standardized exercises designed to improve dexterity of the non-dominant hand. The first exercise, which lasted 10 min each day, was to turn coins over using the index finger and thumb. The second was to put a knot around a screw by holding the knot and screw with the non-dominant and dominant hands, respectively. The third, which lasted 40 min each day, was to write letters. The duration of MT was 60 min each day.

### EEG recording

Electroencephalography (EEG) was recorded for two and a half minutes before and after rTMS-MT in an awake, resting state during which subjects kept their eyes closed. We chose to test subjects with eyes closed to reduce the presence of eye movement and muscle artifacts. Electrode montage and placement was recording to the international 10/10 system. EEG signals were acquired through a 64-channel synamps2 EEG system (Neuroscan, Compumedics, USA). A 64-channel EEG cap was positioned on subjects' head. The reference electrode was at AFz site, whereas the ground electrode was at FCz site. The impedance for all electrodes was kept below 5 kΩ. The activities in the right eye vertical and horizontal electroculogram (vEOG) were registered from two surface electrodes. The EEG data was sampled at a frequency of 1,000 Hz and subsequently processed offline.

### Phase synchronization index

Phase synchronization index (PSI) can quantify neural signal interaction between brain regions, but neglect the effect of instantaneous amplitude. This method has been proven effective in inferring functional connectivity between neural oscillations (Varela et al., 2001; Ito et al., 2005; Wu et al., 2011).

Given an EEG signal *x*(*t*), its analytical signal is defined as:

(1) $$\begin{array}{l} {\text{Z}}_{\text{x}}\left(t\right)=\text{x}\left(t\right)+\text{i}\bar{\text{x}}\left(t\right)={\text{A}}_{\text{x}}^{\text{H}}\left(t\right){\text{e}}^{i\Phi_{\text{x}}^{\text{H}}\left(t\right)} \end{array}$$

Where

(2) $$\begin{array}{l} \bar{x}\left(t\right)=Hx\left(t\right)=\frac{1}{\pi }\overset{+\infty }{\underset{-\infty }{\int }}\frac{x\left(t^{\prime }\right)}{t-t^{\prime }}d\left(t^{\prime }\right) \end{array}$$

is the Hilbert transformation of signal $x\left(t\right).\;{\text{A}}_{\text{x}}^{\text{H}}\left(t\right)$ and $\Phi_{\text{x}}^{\text{H}}\left(t\right)$ are the instantaneous amplitude and phase of signal x(*t*), respectively. Let $\Phi_{\text{x}}^{\text{H}}\left(\text{t}\right)$ and $\Phi_{\text{y}}^{\text{H}}\left(t\right)$ denote the cumulative instantaneous phases of two coupled signals, respectively. If $\Phi_{\text{x}}^{\text{H}}\left(t\right)$ and $\Phi_{\text{y}}^{\text{H}}\left(t\right)$ satisfy.

(3) $$\begin{array}{l} \Phi_{\text{xy}}^{\text{H}}\left(t\right)\equiv |n\Phi_{\text{x}}^{\text{H}}\left(t\right)-m\Phi_{\text{y}}^{\text{H}}\left(t\right)|<const., \end{array}$$

then the two signals x(*t*) and y(*t*) are said to be in *n*:*m* phase synchronization, where const. is a constant, and *n* and *m* are positive integers. Equation (3) implies that the *n*:*m* instantaneous phase difference of signals x(*t*) and y(*t*) is bounded. As mentioned by a number of authors (Quiroga et al., 2000; Quian Quiroga et al., 2002; Angelini et al., 2004; David et al., 2004), 1:1 phase synchronization is the case for most neurophysiological signals. The PSI is defined as:

(4) $$\begin{array}{l} \lambda_{\text{H}}=|{\langle {\text{e}}^{\text{i}\Phi_{\text{xy}}^{\text{H}}\left(t\right)}\rangle }_{t}|=\sqrt{{\langle \cos\Phi_{\text{xy}}^{\text{H}}\left(t\right)\rangle }_{t}^{2}+{\langle \sin\Phi_{\text{xy}}^{\text{H}}\left(t\right)\rangle }_{t}^{2}}, \end{array}$$

where 〈.〉 denotes the average over time. Note that the value of λ_H_ is among [0, 1] with λ_H_ = 1 implying perfect phase synchronization, and λ_H_ = 0 indicating no phase synchronization at all.

### Generating synchronization matrices and graph theory analysis

To evaluate changes in network topologies induced by rTMS-MT, we constructed undirected graphs before and after rTMS-MT. There are 60-channels EEG signals for each subject. For each subject, we could obtain a 60-by-60 matrix for all 60 channels in pairs. Then, for each individual data set, a connectivity graph was formed consisting of 60 nodes (EEG channels) and a set of undirected edges, A (functional connectivity) obtained by applying a correlation threshold, T to the PSI matrix:

(5) $$\begin{array}{l} A_{ij}=\{\begin{array}{cc} 1 & \lambda_{\text{H}}\geq T\\ 0 & \lambda_{\text{H}}<T \end{array}\;(\text{i},\text{j}=1,2,...,60) \end{array}$$

Hence, if the PSI value between a pair of brain regions i and j was greater than the given value T, an edge was said to exist.

In our study, the threshold was set independently before and after rTMS-MT to produce the same mean connectivity degree in each period (over a range of mean connectivity degrees) for each subject and for the grand average across subjects (fixed-density analyses). The fixed-density analyses were performed systematically over a range of thresholds, resulting in networks with average connection densities ranging from two up to 20 connections per node.

The three most commonly evaluated graph theory statistics include the node degree, clustering coefficient, and efficiency. The node degree is defined as the number of edges one node connects with other nodes. The greater the node degree, the more edges this node has, and therefore could exert greater influence on the network. The degree, D of node i is defined as:

(6) $$\begin{array}{l} D_{i}=\overset{N}{\underset{j=1}{\sum }}a_{ij} \end{array}$$

The clustering coefficient evaluates the proportion of neighboring vertices (i.e., vertices directly connected to the node in question) that are connected to each other. Networks with higher clustering are said to have greater local efficiency of information processing and robustness. The clustering coefficient of node i is defined as.

(7) $$\begin{array}{l} C_{i}=\frac{2E_{i}}{k_{i}\left(k_{i}-1\right)} \end{array}$$

Where *k*_*i*_ is the number of neighboring nodes connected with node i, and *k*_*i*_(*k*_*i*_ −1)/2 denotes all possible edges among neighbor nodes of node i.

Node efficiency is the average of the inverse of the shortest path length, and reflects the efficiency of information transmission. The greater the node efficiency, the more efficient the information transfer. The efficiency of node i is defined as:

(8) $$\begin{array}{l} {\text{E}}_{\text{glob}}=\frac{1}{N(N-1)}\sum_{i,\;j\subseteq \;V,i\neq j}\frac{1}{{\text{d}}_{\text{ij}}} \end{array}$$

### Partition of all electrodes

**Figure 2:**
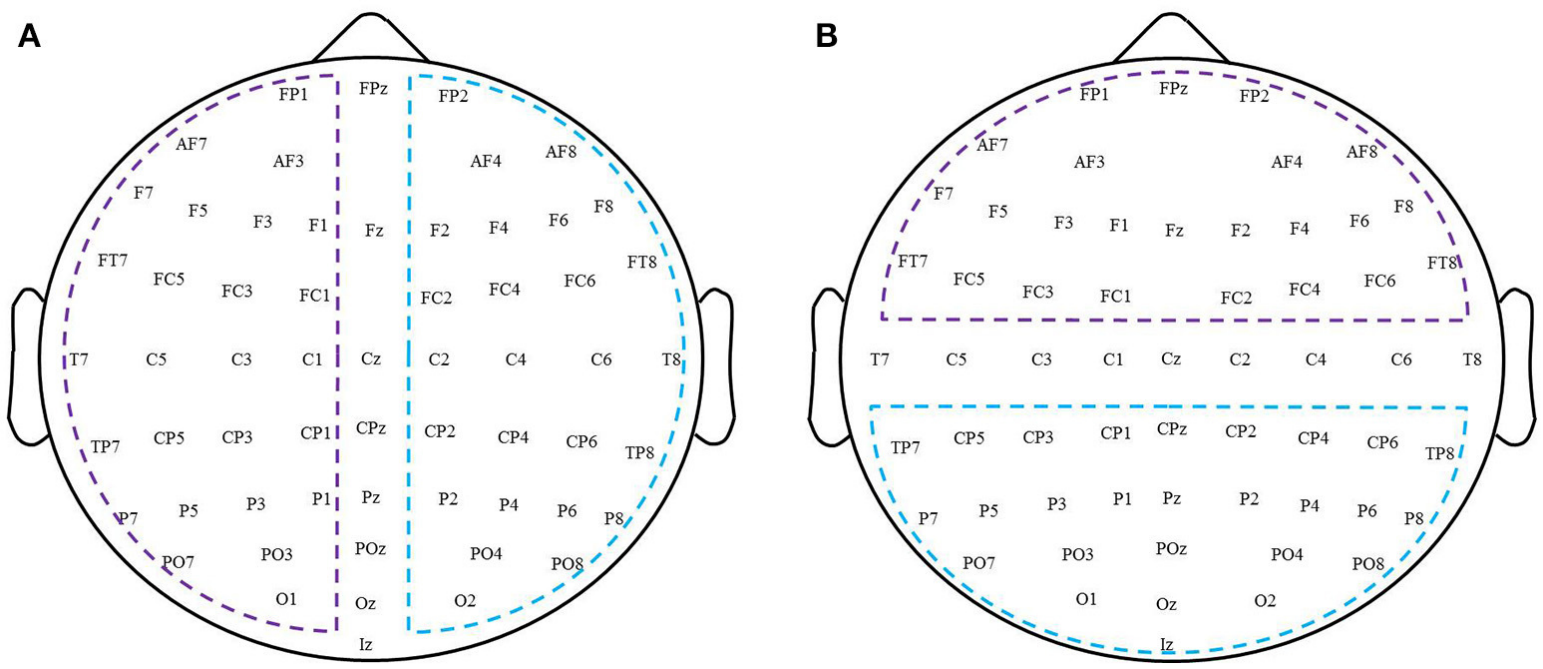
A schematic diagram of partition for all electrodes. **(A)** Left-right hemispheric. **(B)** Anterior-posterior region.

To quantitatively examine patterns of changes in functional connectivity during rTMS-MT, we grouped connections along the anterior-posterior and right-left axes, and performed statistical tests on these groups. Figure 2 shows the schematic diagram of partition for all the electrodes. In the anterior-posterior analysis (Figure 2A), the EEG channels were first partitioned into anterior, central, or posterior regions. The anterior region consisted of Fp1, Fp2, F7, F3, Fz, F4, F8, FC5, FC1, FC2, and FC6. The central region consisted of T7, C5, C3, C1, Cz, C2, C4, and T8. The posterior region consisted of CP5, CP1, CP2, CP6, P7, P3, Pz, P4, P8, O1, Oz, and O2. If both electrodes were anterior, or if one was anterior and the other central, then the connection between the two was designated as “anterior.” If both electrodes were posterior, or if one was posterior and the other central, the connection was “posterior.” Connections where one electrode was from the anterior region and the other from the posterior region were defined as “interregional.” Connections between two central electrodes were ignored during subsequent analyses.

To analyze changes in connectivity induced by rTMS-MT within a hemisphere, EEG channels were first partitioned into left hemispheric, right hemispheric, and midline subsets according to standard EEG convention (left hemispheric—Fp1, F7, F3, FC5, FC1, T7, C5, C3, C1, CP5, CP1, P7, P3, and O1; right hemispheric—Fp2, F8, F4, FC6, FC2, T8, C4, C2, CP6, CP2, P8, P4, and O2; midline—Fz, Cz, and Oz; Figure 2B). If both electrodes were right hemispheric, or if one was right hemispheric and the other midline, the connection between the two was designated as “right intra-hemispheric” connection. If both electrodes were left hemispheric, or if one was left hemispheric and the other midline, the connection was designated as a “left intra-hemispheric” connection. If one electrode was right hemispheric and the other left hemispheric, the connection was designated as “inter-hemispheric.” Connections between two midline electrodes were ignored. Subsequent statistical analysis was conducted following the same approach as described above for the connections grouped according to regions.

### Data processing and statistical analysis

Electroencephalography (EEG) data was processed offline using Matlab (version 10.0) and EEGLAB toolbox (version 13.0). The preprocessing stage of the EEG signals is required before further analysis. 1 min data without apparent artifacts (such as EMG and visible drift) were selected manually from each volunteer's EEG recording. The data were first down-sampled from 1,000 to 250 Hz. And then all channels were rereferenced to bilateral mastoid. The bandpass filter was used to extract alpha band signals (8–13 Hz). The alpha band signals were then split into 5-s, non-overlapping epochs in the following steps of feature extraction. 12 epochs of artifact-free EEG data for each subject were obtained finally.

Statistical differences before and after rTMS-MT were tested with a nonparametric rank sum test. Pearson's correlation was used to assess the relation between PSI and hand dexterity. All data were presented as mean ± standard deviation. A *p* < 0.05 was considered statistically significant, except the PSI functional connectivity (*p* < 0.005). All statistical analyses were performed using matlab software (Mathworks Inc., US).

## Results

### Behavioural results

**Figure 3:**
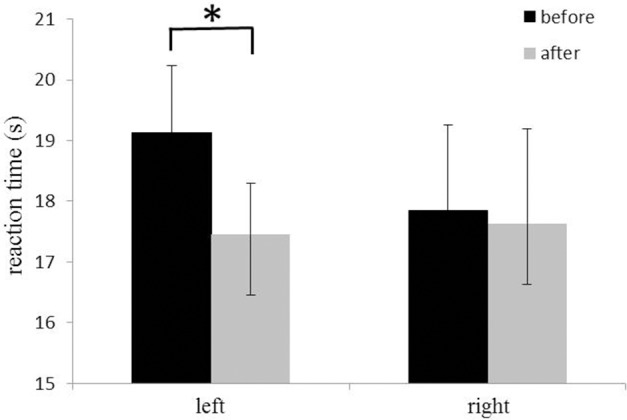
Changes in reaction time of Nine-Hole Peg test of hands. The reaction time of the left hand (non-dominant hand) decreased significantly after rTMS-MT (^*^p < 0.05).

The 14-days rTMS-MT resulted in a significant decrease in reaction time for left hand (rTMS-MT group, before: 19.1 ± 1.1 s, after: 17.5 ± 1.4 s, *p* = 0.001; Figure 3), but not right hand dexterity (before: 17.9 ± 0.8 s, after: 17.6 ± 1.6 s, *p* = 0.281; Figure 3). The data suggested a significant improvement of motor performance for the left hand (non-dominant hand).

### Functional connectivity

**Figure 4:**
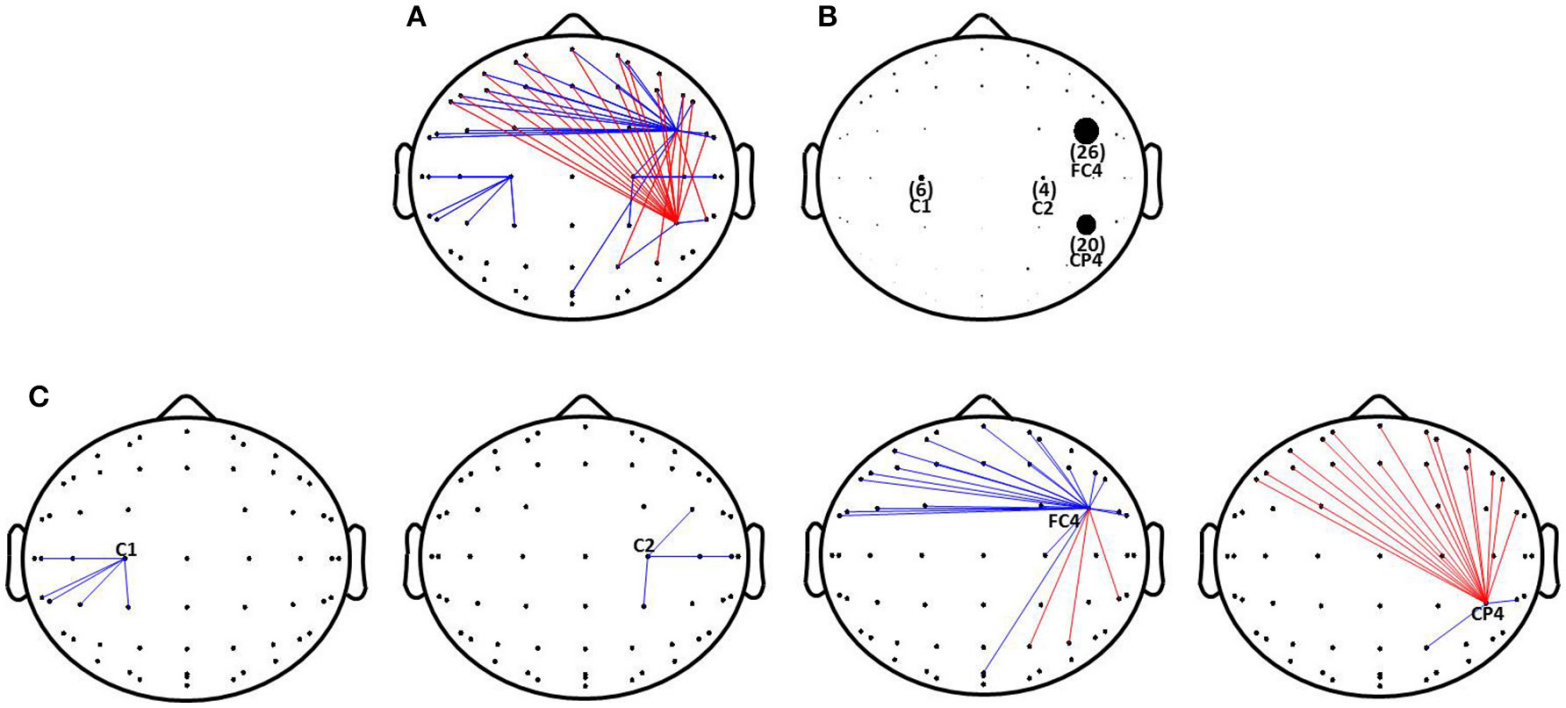
Changes of functional connectivity in alpha frequency band induced by rTMS-MT. Significant decrease and increase in functional connectivity are designated by blue and red lines, respectively (^*^p < 0.005). **(A)** Changes of functional connectivity. **(B)** The number of functional connectivity induced by rTMS-MT. **(C)** Changes of functional connectivity in C1, C2, FC4, and CP4 electrodes.

A 60 × 60 channel matrix, consisting of the PSI values for each electrode pair, was obtained for each subject before and after rTMS-MT, and significant changes in connectivity were assessed for all subjects at a significance level of *p* < 0.005. Figure 4A shows the functional connectivity with significant difference before and after rTMS-MT. The results suggest that the functional connectivity in the alpha frequency band was changed by the 14-days rTMS-MT, and the changes were region-specific.

In order to clearly show the changes in functional connectivity, we calculated the number of connections with significant differences (*p* < 0.005), and expressed them with different-size dots (Figure 4B). rTMS-MT might mainly affect brain regions related to motor function in the right hemisphere (non-dominant hemisphere), especially FC4 and CP4. There was a decrease in strength of functional connectivity between FC4 and the frontal region, and an increase between CP4 and the frontal region (Figure 4C). Furthermore, C1 and C2 were also slightly influenced. There was a decrease between C1, C2, and parietal region of the ipsilateral hemisphere (Figure 4C).

### Network topology

**Figure 5:**
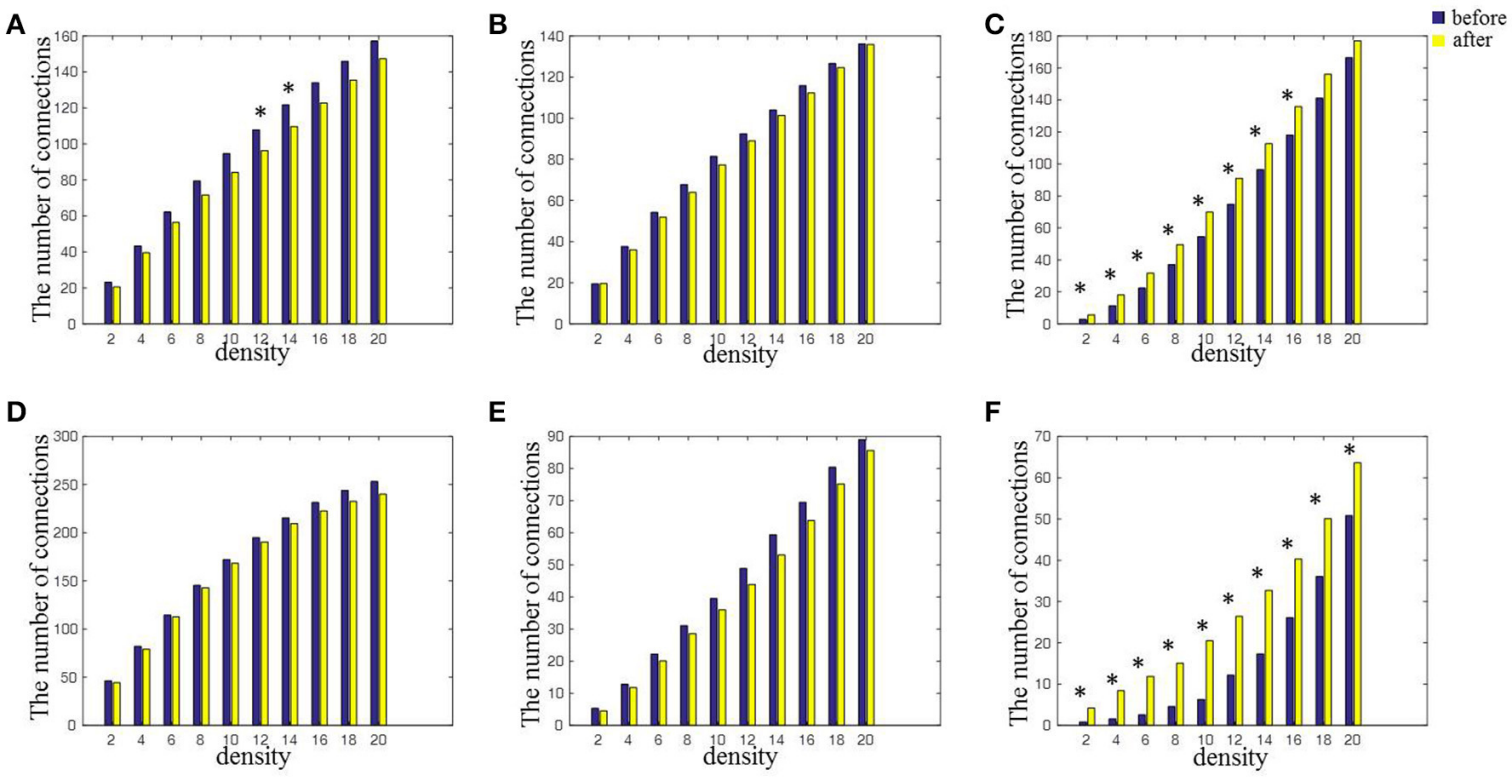
The changes in connectivity induced by rTMS-MT for alpha frequency band between intra-hemispheric regions, inter-hemispheric regions, intra-regions, and inter-regions (^*^p < 0.05). **(A)** Left-hemisphere. **(B)** Right-hemisphere. **(C)** Inter-hemisphere. **(D)** Anterior-region. **(E)** Posterior-region. **(F)** Inter-region.

Figure 5 shows the number of connections in intra-hemispheres, inter-hemispheres, intra-regions, and inter-regions with a mean connectivity degree from 2 to 20 before and after rTMS-MT. It could be observed that the number of connections decreased within intra-hemispheres and intra-regions, and increased within inter-hemispheres and inter-regions in all connectivity degrees. A nonparametric rank sum test was performed (^*^p < 0.05). Significant differences were found within inter-hemispheres, left intra-hemispheres (K = from 12 to 14), and inter-regions (K = from 2 to 20) before and after rTMS-MT.

### Network characteristics

**Figure 6:**
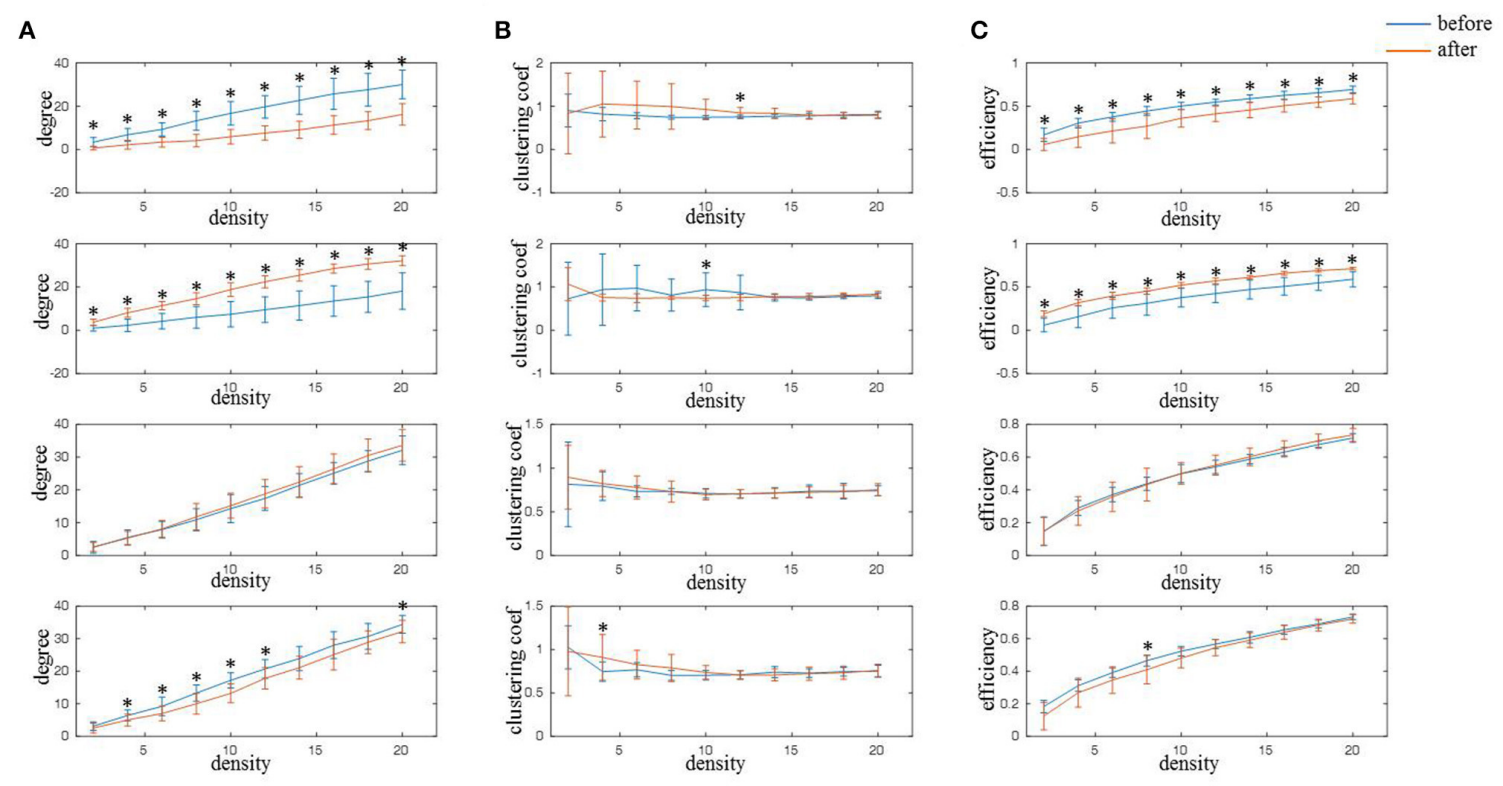
The changes of network characteristics in alpha frequency band for FC4, CP4, C1, and C2 electrodes induced by rTMS-MT (K = from 2 to 20) (^*^p < 0.05). Blue and red lines represent the network characteristics before and after rTMS-MT, respectively. **(A)** Node degree. **(B)** Clustering coefficient. **(C)** Node efficiency.

From the results of functional connectivity, it can be inferred that rTMS-MT mainly induced changes at nodes FC4, CP4, C2, and C1 in alpha band. We calculated the network characteristics including node degree, clustering coefficient, and efficiency of nodes with different connectivity degrees (K = from 2 to 20), and the results are shown in Figure 6. It could be observed that both node degree and efficiency decreased at FC4 (K = from 2 to 20) and C1 (K = from 4 to 12, 20 for node degree; K = 8 for node efficiency), but increased significantly at CP4 (K = from 2 to 20) in the variable-densities networks. The clustering coefficient was not significantly different before and after rTMS-MT.

### Relationship of functional connectivity and behavioral results

We calculated the number of significant functional connections between the FC4, CP4, C1, C2, and other electrodes before and after rTMS-MT. The relationship of functional connectivity and reaction time of Nine-Hole Peg Test for the -dominant hand was calculated using Pearson's correlation, and the results are shown in Table 1. We found strong positive correlation between changes in functional connectivity and motor function at FC4 (*r* = 0.712, *p* = 0.048). We also found correlations between changes in functional connectivity and motor function at C1(*r* = 0.672, *p* = 0.068) and C2 (*r* = 0.594 *p* = 0.121) respectively, even if there were not significant.

**Table 1:** Relationship of functional connectivity and reaction time of Nine-Hole Peg Test for non-dominant hand.

	**FC4**	**CP4**	**C2**	**C1**
correlation	**0.712**	0.371	0.594	0.672
*P*	**0.048**	0.365	0.121	0.068

## Discussion

It had been reported that rTMS-MT could improve motor performance, however, studies on modulation of rTMS-MT on brain functional connectivity were still scarce. In our study, we evaluated the changes of functional connectivity in alpha frequency band induced by 14-days of rTMS-MT. We found that rTMS-MT significantly improved hand dexterity. The functional connectivity and network characteristics of cortices related to motor function in non-dominant hemisphere changed, and the number of connections mainly increased within inter-hemispheres and inter-regions. In other words, the rTMS-MT had an obvious impact on long connections of brain regions. Here we showed for the first time that alternations of rTMS-MT could reflect in changes of brain functional connectivity and topological functional organization.

### rTMS-MT improved motor performance

It is important to optimize motor function and improve motor dexterity, especially for post-stroke patients. In recent years, researchers have tested motor performance and corticospinal excitability and found greater behavioral and neurophysiologic outcomes induced by rTMS-MT than rTMS or MT alone (Emara et al., 2010; Avenanti et al., 2012; Kakuda et al., 2012, 2016; Lüdemann-Podubecká et al., 2015). In our study, we measured hand dexterity using Nine-Hole Peg Test and also found significant improvement in motor performance after 14-days rTMS-MT (Figure 3), which demonstrated the effectiveness of rTMS-MT in improvement of motor function.

### rTMS-MT modulated the functional connectivity in non-dominant hemisphere

In our study, we evaluated the connectivity changes induced by rTMS-MT, and found it modulated the functional connectivity in non-dominant hemisphere and in a region-specific way (Figure 4). The 14-days MT alone didn't appear those changes (Figure S2) when the significance level was *p* < 0.005 which was the same as the rTMS-MT group. Subsequently, we calculated the significant changes induced by MT at a significance level of *p* < 0.05 (Figure S3). The results suggested that the functional connectivity of motor cortex in non-dominant hemisphere was also changed. Therefore, the effects of rTMS-MT on functional connectivity were greater than MT alone. In rTMS-MT group, rTMS might play a meaningful role. The changes induced by rTMS-MT were not entirely caused by MT alone.

Besides, the results in our study were inconsistent with the effects of rTMS alone. rTMS could modulated the functional connectivity of widespread cortical networks and activates neural circuits in a non-specific way, although the modulation of rTMS varied as a function of the particular rTMS protocol applied and the frequency band analyzed (Strens et al., 2002; Chen et al., 2003; Oliviero et al., 2003; Fuggetta et al., 2008; Bolognini et al., 2009; Shafi et al., 2014). Especially, the connectivity changes between cortical and cortical regions in stimulated hemisphere were a consistent finding. In our study, we also found widespread changes of functional connectivity induced by rTMS-MT. However, the changes in regions induced by rTMS-MT were mainly observed in non-dominant hemisphere (not stimulated hemisphere) related to motor function, especially in premotor and parietal cortices. So, the changes induced by rTMS-MT were not simply caused by rTMS alone.

Taken together, our study represents the first analysis of network-level connectivity changes induced by rTMS-MT. The results suggested that changes induced by rTMS-MT in our study would be in a region-specific way, and might not come from the rTMS or MT alone, and also not linear superposition of rTMS and MT. Motor training could guide the activation of specific neural networks associated with the desired behavior (Bolognini et al., 2009). It was speculated that practice of a motor task might be more effective at using the neural mechanisms sub-serving training-dependent plastic changes if pertinent areas of cortex were facilitated (Ward and Cohen, 2004). The results in our study supported this assumption. rTMS-MT could enhance the functional connectivity induced by MT in a task-specific way.

### rTMS-MT changed the functional connectivity and network characteristics related to motor function

We tested changes in functional connectivity induced by rTMS-MT, and found that the alpha band functional connectivity was modulated by 1 Hz rTMS of the dominant hemisphere combined with MT by the non-dominant hand over 14-days. The oscillation at alpha frequency band is the intrinsic rhythms in resting state with eyes closed, which can reflect the state of cortical information processing during motor tasks (Babiloni et al., 2010). Besides, Dubovik et al. (2012) studied changes of synchrony of electrical oscillations in neural networks after stroke, and found the alpha band functional connectivity decreased in ipsilesional central electrodes, which was correlated with motor task performance. Therefore, the functional connectivity at alpha band might be important to improvement of motor function in stroke, and the connection changes induced by rTMS-MT at alpha band oscillation might be the reason of motor improvement.

In our study, the changes of functional connectivity were especially found in the premotor (FC4) and parietal cortices (CP4) in the non-dominant hemisphere. Moreover, the connections of primary motor cortices (C1 and C2) in both hemispheres were also slightly changed (Figures 4A,B). We found a decrease in functional connectivity between premotor and frontal cortices, and an increase between the parietal and frontal cortices (Figure 4C). Motor skill learning involves many processes such as motor planning, motor controlling, spatial orientation, and motor output, and is associated with a variety of cortical activities, including the primary motor, posterior parietal, and premotor cortices, and the functional connectivity between these cortices (Hardwick et al., 2013). Researchers have reported that the connectivity of the resting cortex could predict motor skills, and the connectivity between the frontal and parietal cortices were significantly correlated with the ability to perform motor skills (Wu et al., 2014). Consistent with reported results, we also found that rTMS-MT changed the connectivity between the parietal and frontal cortex in the resting state, along with improvement of motor performance (Figure 4), which was related to the role of the frontal and parietal cortex in motor skills learning (Ma et al., 2011; Wu et al., 2014). Furthermore, the increased connections of premotor cortex were the symbol of inefficient motor system (Ward, 2006). Our study also revealed decreased connections and functional coupling between premotor and frontal cortex, which might imply that rTMS-MT modulated those brain regions to enhance the efficient of the motor system.

We subsequently thresholded the functional connectivity to construct undirected graphs. Threshold can influence the network density. Higher threshold can decrease the number of network connections leading to low network density. In contrast, low threshold can increase the number of network connections leading to high network density. In this study, in order to produce the same network density for all subjects before and after rTMS-MT, the threshold was set independently. Because there is no uniform standard for threshold selection, we assessed the network characteristics induced by rTMS-MT in different connectivity degrees (K = from 2 to 20). The results demonstrated changes in node degree and efficiency of FC4 and CP4 electrodes after rTMS-MT in almost all connectivity degrees (Figure 6). More than other electrodes, the degree and efficiency of FC4 electrode significantly decreased, which suggested the importance of this region became weaker and the information transmission became slower in brain network. Besides, the degree and efficiency of CP4 electrode showed opposite results. These results were consistent with those of functional connectivity. Therefore, rTMS-MT modulated the neural circuits of motor skill learning and increased the efficiency of the motor system in resting state.

Our experimental design was based on the competition model of the two brain hemispheres. According to the purpose of the experimental, rTMS was performed to dominant hemisphere, and non-dominant hand executed the MT. However, we couldn't found remarkable changes of functional connectivity between central and central regions in both hemispheres. We speculated that the changes of brain activity between central and central regions induced by rTMS-MT mightn't be reflected by functional connectivity based on PSI. Grefkes et al. (2008), Grefkes and Fink (2011) studied the changes of connectivity using resting state fMRI in stroke, and didn't found the changes of central-central connectivity, compare with healthy person, which demonstrated the competition between interhemisphere might not be reflected by the central-central connectivity in resting state. Therefore, while we focused on the changes of functional connectivity in resting state induced by rTMS-MT, we couldn't found changes of central-central between interhmisphere. In the course of our experiment, we also assessed the excitability changes of corticospinal tract through testing the motor evoked potentials (MEP), the result was shown in Supplementary Material 2 (Figure S6). We found the MEP amplitudes of non-dominant hand increased and the dominant hand didn't change, which indicated change of central and central regions and reflected the competition model to a certain degree.

### rTMS-MT could modulate network topology

In our study, although rTMS, and MT were applied to the left motor cortex (dominant hemisphere) and left hand (non-dominant hemisphere), respectively, we found changes in connections mainly occurred within inter-hemisphere at almost all network densities, rather than left or right hemisphere (Figure 5). This was suggestion that the rTMS-MT increased the connections and the information transmission between inter-hemisphere. It has been reported that homologous and heterologous regions of inter-hemispheric regions were connected through a large number of nerve fibers in the corpus callosum, which supported information transmission and functional integration of the two hemispheres (Jing and Takigawa, 2000; Paul et al., 2007). Therefore, the increasing connections were consistent with the presence of significant transcallosal anatomic connectivity between homologous areas.

In addition, the increased connections within inter-hemisphere induced by rTMS-MT might be a critical factor for improvement of motor function in stroke. Previous studies utilizing a variety of neuroimaging techniques had demonstrated that there were widespread cortical networks abnormalities after stroke, which were reflected in not only in lesion regions but also among the interactions among cortical areas distant from the lesion (Honey and Sporns, 2008; Carter et al., 2010; Grefkes et al., 2010; Wang et al., 2010). Furthermore, the consistent finding was that inhibition of inter-hemisphere was changed in patients with motor dysfunctions, and the improvement of motor performance was shown to be correlated with restitution of inter-hemispheric connectivity (Ward and Cohen, 2004; He et al., 2007; Carter et al., 2010; van Meer et al., 2010). It was helpful for recovery of motor function to enhanced inter-hemispheric connectivity (Grefkes et al., 2010). Therefore, the effects of rTMS-MT on inter-hemispheric connectivity might be another reason for improvement of motor performance.

## Limitations

There were certain limitations with our study. First, the number of subjects was small (10 subjects were included in the final analysis) and there was no blank control group. Future studies with more subjects and/or a group of blank control subjects could help provide greater insight into resting state network changes associated with the effects of rTMS-MT. Furthermore, all subjects in this study were healthy people. Although, motor performance enhancement is important for healthy individuals, it is more meaningful for studies on patients with motor dysfunctions. Moreover, brain activity influenced by rTMS-MT on patients might be different from that on the healthy person, so it is necessary in the next step to study the effect of rTMS-MT on functional connectivity of patients, especially people with stroke. An additional consideration is that the design of this study did not allow dissection of effects induced by each intervention (i.e., rTMS or MT) on functional connectivity. In our study, all volunteers are all healthy. The tasks were selected to improve the hand dexterity. We didn't test other function, such as hand grip power and pinch power and so on, which might be a further limitation of this study. Notwithstanding these limitations, the results in this study were helpful in understanding the modulation of rTMS-MT on functional connectivity. Meanwhile, the methods in this study could in future be used to evaluate changes of brain activities induced by rTMS-MT in stroke patients.

## Conclusion

In summary, our findings suggest that rTMS-MT could improve motor function and induce significant changes in functional connectivity and network characteristics in regions related to motor functions. The network analysis techniques might be a promising method to study how changes of information integration occur between regions induced by rTMS-MT. The findings in this paper could be helpful in understanding the impact of rTMS-MT on brain activity. The methods and results in this study could be taken as reference for future research of the effects of rTMS-MT in stroke patients.

## Author contributions

J-NJ, YL, and FJ were responsible for the design of the work, and completed the experiment for acquiring the EEG data. After that, J-NJ, XW, and Z-PL completed the analysis and the interpretation of the data. All authors participated in drafting the manuscript. J-NJ, Z-PL, and TY were responsible for revising the important intellectual content involved in the article and approved the final version of the article.

### Conflict of interest statement

The authors declare that the research was conducted in the absence of any commercial or financial relationships that could be construed as a potential conflict of interest.
