# Supplementary material for: The Effects of rTMS Combined with Motor Training on Functional Connectivity in Alpha Frequency Band
Source: Front Behav Neurosci. 2017 Nov 29;11:234. doi: 10.3389/fnbeh.2017.00234 (PMC5712595; doi:10.3389/fnbeh.2017.00234)
Supplement: Supplementary file 1 [file Presentation1.PDF]

# Supplementary Material 1

## The effects of Motor Training on Functional Connectivity

## 1 Material and Methods

### 1.1 Subjects

We recruited 9 healthy volunteers (7 men and 2 women; mean age 26.7±2.70 years) to perform motor training (MT) alone. None of volunteers suffered from any significant neurological disorder, and took any medication in the 2weeks before their participation in the experiment. All subjects were right handed, according to the Edinburgh handedness inventory. The study was performed according to the Declaration of Helsinki and approved by the Ethics Committee of Institute of Biomedical Engineering, Chinese Academy of Medical Sciences& Peking Union Medical College. All subjects provided informed consent prior to inclusion in the study.

### 1.2 Procedure

In this study, the MT consisted of 3 standardized exercises designed to improve dexterity of the non-dominant hand. The modes of MT were same as the MT in rTMS-MT. **Figure S1** showed the experimental paradigm. The subjects performed MT for 14 days. The Nine-Hole Peg Test was performed to assess the dexterity of bilateral hands. The reaction time was recorded, and resting EEG with eyes closed was obtained before and after MT.

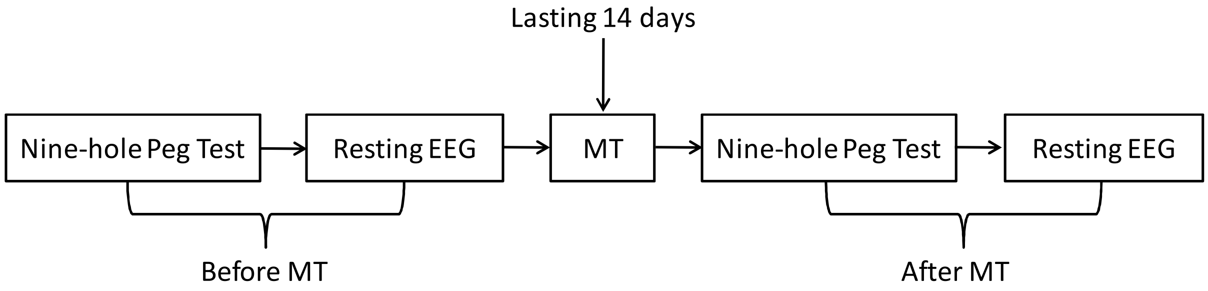


**Figure S1** Experimental paradigm. The non-dominant hand performed MT during 14 days. Nine-Hole Peg Test and resting state EEG with eyes closed were recorded before and after MT.

### 1.3 EEG recording and analyzing were same as the rTMS-MT group

## 2 Results

### 2.1 Behavioural results

The 14-days MT resulted in a significant decrease of reaction time for left hand dexterity (before: 20.6±2.2s, after: 19.7±2.1s, p=0.011), but didn't for right hand (before: 19.4±2.2s, after: 18.9±1.6s, p=0.304). Those data suggested a significant improvement of motor performance for left hand (non-dominant hand).

### 2.2 Functional connectivity

A 60 × 60 channel matrix, consisting of the phase synchronization index (PSI) values for each electrode pair, was obtained for each subject before and after MT, and significant changes in connectivity were assessed for all subjects at a significance level of $p < 0.005$, as shown in **Figure S2**. The functional connectivity was barely changed, only between Pz and CP1 electrodes. Subsequently, we calculated the significant changes of connectivity at a significance level of $p<0.05$, as shown in **Figure S3**. The results suggested that functional connectivity in alpha frequency band was changes by 14-days MT (**Figure S3A**), especially Pz and C4 electrodes (**Figure S3B**). The functional connectivity between C4 and frontal region decreased, and between Pz and central region in two hemisphere increased (**Figure S3C**).

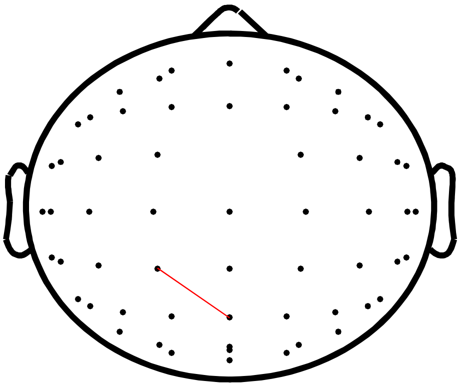

**Figure S2** Changes of functional connectivity in alpha frequency band induced by MT. The significance level is **p<0.005.** Significant increase in functional connectivity was designated by red lines,

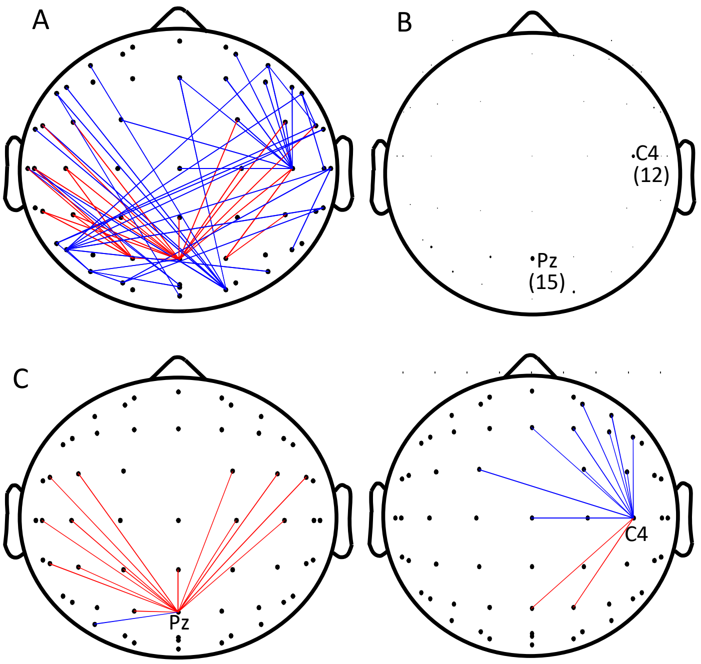


**Figure S3** Changes of functional connectivity in alpha frequency band induced by MT. The significance level is **p<0.05.** Significant decrease and increase in functional connectivity were designated by blue and red lines, respectively. **(A)** Changes of functional connectivity. **(B)** The number of functional connectivity induced by MT. **(C)** Changes of functional connectivity in Pz and C4 electrodes.

## 2.3 Network Topology

Figure S4 showed the number of connections in intra-hemispheres, inter-hemispheres, intra-region and inter-region with a mean connectivity degree from two to twenty before and after MT. The number of connections were increase within inter-hemispheres, even if it was not significant. The number of connections were decrease significantly in low network density (K=2:6) (*$p < 0.05$).

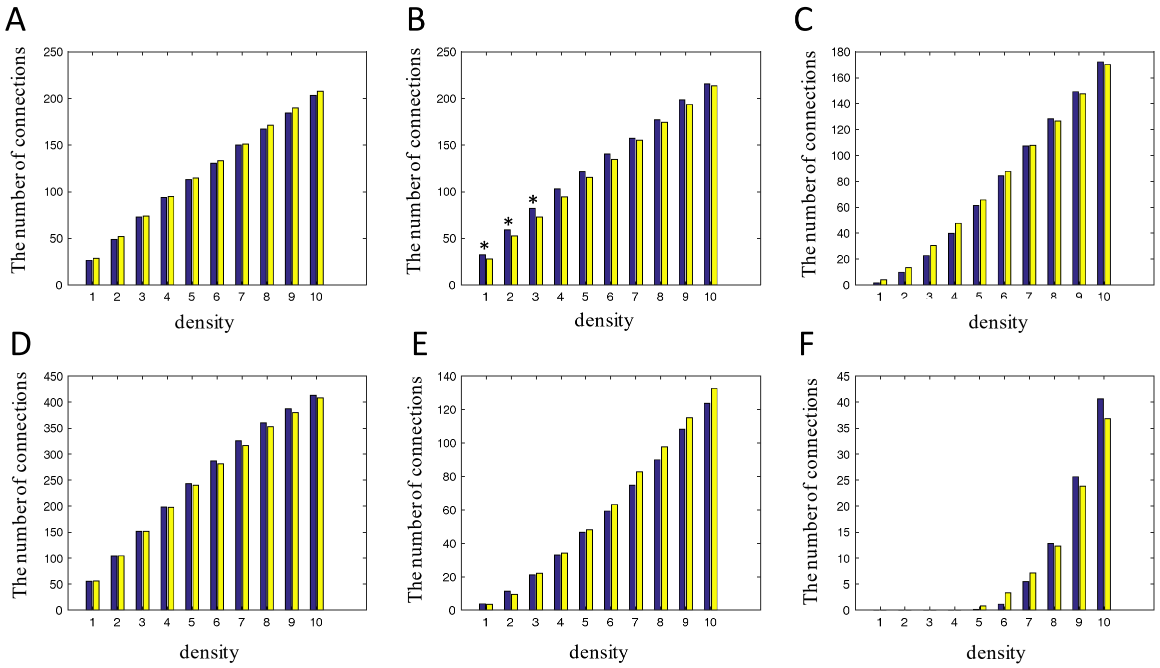


**Figure S4** The changes in connectivity induced by MT for alpha frequency band between intra-hemispheric regions, inter-hemispheric regions, intra-regions, and inter-regions (*p < 0.05). (A) Left-hemisphere. (B) Right-hemisphere. (C) Inter-hemisphere. (D) Anterior-region. (E) Posterior-region. (F) Inter-region.

## 2.4 Network Characteristics

From the results of functional connectivity, it can be inferred that MT mainly induced changes at nodes C4 and Pz in alpha band. We calculated the network characteristics of those nodes with different connectivity degree (K=2:20), including node degree, clustering coefficient and efficiency, as shown in **Figure S5**. The node degree and efficiency showed a reducing trend at C4 electrode, and a rising trend at Pz electrode. But it was significant only in limited network density.

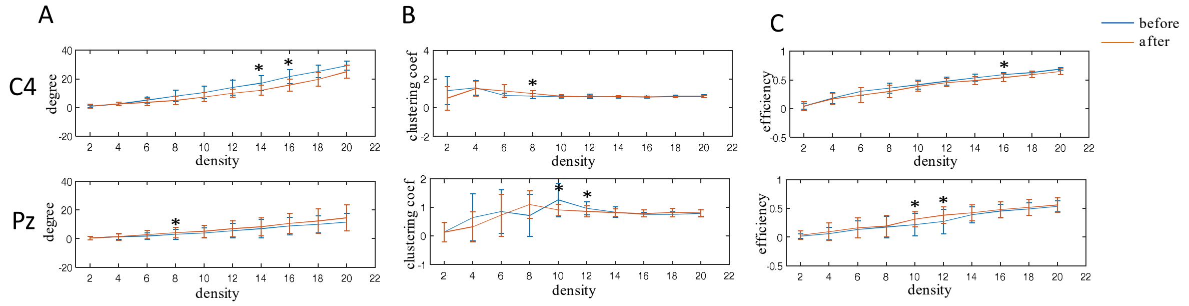


**Figure S5** The changes of network characteristic in alpha frequency band for C4 and Pz electrodes induced by MT (K=from 2 to 20) (*p<0.05). Blue and red lines represent the network characteristics before and after rTMS-MT, respectively. (A) Node degree. (B) Clustering coefficient. (C) Node efficiency.
