# Supplementary material for: The Effects of rTMS Combined with Motor Training on Functional Connectivity in Alpha Frequency Band
Source: Front Behav Neurosci. 2017 Nov 29;11:234. doi: 10.3389/fnbeh.2017.00234 (PMC5712595; doi:10.3389/fnbeh.2017.00234)
Supplement: Supplementary file 2 [file Presentation2.PDF]

Supplementary Material 2

The effects of rTMS-MT on corticospinal tract excitability

In our study, we tested the changes of corticospinal tract excitability induced by rTMS-MT. Single TMS was performed over primary motor cortex (M1). The intensity of single TMS was set at 110%RMT. A total 30 pulses were carried out. The pulse interval was 5s. We recorded the motor evoked potentials (MEPs) before and after rTMS-MT, and the result were shown in Figure S6. It can be observed that the amplitude of MEPs increased for left hand (don-dominant hand) after rTMS-MT (before: 186.32±122.92 μ V, after: 225.36±96.08 μ V, p=0.042), but didn't for right hand (dominant hand) (before: 206.99±80.26 μ V, after: 251.21±105.85ms, p=0.197). Those data suggested a significant improvement of corticospinal tract excitability for non-dominant hemisphere.

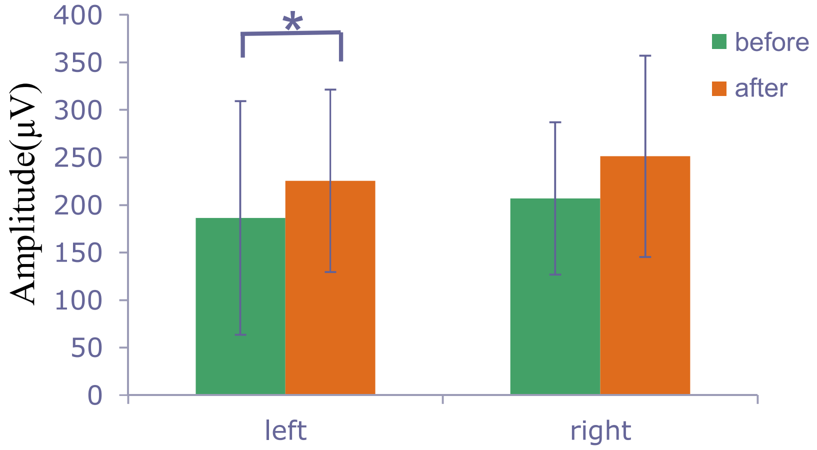

Figure S6 the changes of MEP induced by rTMS-MT
